# Supplementary material for: End Sequence Analysis Toolkit (ESAT) expands the extractable information from single-cell RNA-seq data
Source: Genome Res. 2016 Oct;26(10):1397–410. doi: 10.1101/gr.207902.116 (PMC5052061; doi:10.1101/gr.207902.116)
Supplement: Supplemental Material [file supp_gr.207902.116_Supp_Materials.tar.gz › ESAT-esat_v0.1/doc/html/classbroad_1_1core_1_1overlaputils_1_1_gene_set_intersect-members.html]

ESAT: Member List


ESAT


- Main Page
- Related Pages
- Packages
- Classes
- Files

- Class List
- Class Index
- Class Hierarchy
- Class Members

All Classes Namespaces Files Functions Variables Enumerator Pages

- broad
- core
- overlaputils
- GeneSetIntersect

broad.core.overlaputils.GeneSetIntersect Member List

This is the complete list of members for broad.core.overlaputils.GeneSetIntersect, including all inherited members.

|  |  |  |
| --- | --- | --- |
| countOverlappers(Map< String, Collection< Gene >> regionsToCount, Map< String, Collection< Gene >> regionsToOverlapWith) | broad.core.overlaputils.GeneSetIntersect | static |
| countOverlappers(Map< String, Collection< Gene >> regionsToCount, Map< String, Collection< Gene >> regionsToOverlapWith, Map< String, Collection< Gene >> excludeSet) | broad.core.overlaputils.GeneSetIntersect | static |
| getOverlap(Map< String, Collection< Gene >> genes, Map< String, Collection< Gene >> intersectSet) | broad.core.overlaputils.GeneSetIntersect | static |
| getOverlap(Map< String, Collection< Gene >> genes, Map< String, Collection< Gene >> intersectSet, boolean verbose) | broad.core.overlaputils.GeneSetIntersect | static |
| getOverlap(Map< String, Collection< Gene >> genes, Map< String, Collection< Gene >> intersectSet, Map< String, Collection< Gene >> excludeSet) | broad.core.overlaputils.GeneSetIntersect | static |
| getOverlap(Map< String, Collection< Gene >> genes, Map< String, Collection< Gene >> intersectSet, Map< String, Collection< Gene >> excludeSet, boolean verbose) | broad.core.overlaputils.GeneSetIntersect | static |
| getOverlapWithRandomizedPositions(Map< String, Collection< Gene >> regions, Map< String, Collection< Gene >> intersectGenes) | broad.core.overlaputils.GeneSetIntersect | static |
| getOverlapWithRandomizedPositions(Map< String, Collection< Gene >> regions, Map< String, Collection< Gene >> intersectGenes, Map< String, Collection< Gene >> excludeFeatures) | broad.core.overlaputils.GeneSetIntersect | static |
| main(String[] args) | broad.core.overlaputils.GeneSetIntersect | static |
| mapGenesToOverlappers(Map< String, Collection< Gene >> genes, Map< String, Collection< Gene >> intersectSet) | broad.core.overlaputils.GeneSetIntersect | static |
| numOverlappingBases(Map< String, Collection< Gene >> genes, Map< String, Collection< Gene >> intersectSet) | broad.core.overlaputils.GeneSetIntersect | static |
| numOverlappingBases(Map< String, Collection< Gene >> genes, Map< String, Collection< Gene >> intersectSet, Map< String, Collection< Gene >> excludeSet) | broad.core.overlaputils.GeneSetIntersect | static |
| numOverlappingGenes(Map< String, Collection< Gene >> genes, Map< String, Collection< Gene >> intersectSet) | broad.core.overlaputils.GeneSetIntersect | static |
| numOverlappingGenes(Map< String, Collection< Gene >> genes, Map< String, Collection< Gene >> intersectSet, Map< String, Collection< Gene >> excludeGenes) | broad.core.overlaputils.GeneSetIntersect | static |
| writeGeneralStats(String geneFile, String intersectFile1, String intersectFile2, String excludeFile, String outFile) | broad.core.overlaputils.GeneSetIntersect | static |
| writeOverlap(String geneFile, String intersectFile, String outFile, boolean randomizePositionsWithinEachGene) | broad.core.overlaputils.GeneSetIntersect | static |
| writeOverlap(String geneFile, String intersectFile, String excludeFile, String outFile, boolean randomizePositionsWithinEachGene) | broad.core.overlaputils.GeneSetIntersect | static |
| writeOverlap(Map< String, Collection< Gene >> genes, Map< String, Collection< Gene >> intersectSet, String outFile, boolean randomizePositionsWithinEachGene) | broad.core.overlaputils.GeneSetIntersect | static |
| writeOverlap(Map< String, Collection< Gene >> genes, Map< String, Collection< Gene >> intersectSet, Map< String, Collection< Gene >> excludeSet, String outFile, boolean randomizePositionsWithinEachGene) | broad.core.overlaputils.GeneSetIntersect | static |


---

Generated on Fri Aug 15 2014 15:10:55 for ESAT by  

 1.8.7
